# Clinical Burden of Chronic Obstructive Pulmonary Disease in Patients with Suboptimal Peak Inspiratory Flow

**DOI:** 10.1155/2024/8034923

**Published:** 2024-03-22

**Authors:** Jill A. Ohar, Donald A. Mahler, Gabrielle N. Davis, David A. Lombardi, Edmund J. Moran, Glenn D. Crater

**Affiliations:** ^1^Section of Pulmonary, Critical Care, Allergy, and Immunologic Diseases, Wake Forest School of Medicine, Winston-Salem, NC, USA; ^2^Geisel School of Medicine at Dartmouth, Hanover, NH, USA; ^3^Valley Regional Hospital, Claremont, NH, USA; ^4^Theravance Biopharma US, Inc., South San Francisco, CA, USA

## Abstract

**Introduction:**

Many patients with chronic obstructive pulmonary disease (COPD) may derive inadequate benefit from dry powder inhalers (DPIs) because of suboptimal peak inspiratory flow (sPIF).

**Objectives:**

To assess the clinical burden of COPD by characterizing the clinical characteristics of participants with sPIF against medium-low resistance DPIs versus those with optimal PIF (oPIF) from two phase 3 clinical trials.

**Methods:**

Baseline data were collected from two randomized, controlled, phase 3 trials (NCT03095456; NCT02518139) in participants with moderate-to-severe COPD. oPIF (60 L/min) against the medium-low resistance DPIs was used as the threshold for defining the PIF subgroups (<60 L/min (sPIF) vs ≥60 L/min (oPIF)).

**Results:**

Most participants included in this analysis were White (92%) and male (63%); the mean (range) age was 65 (43–87) years. Participants with sPIF had significantly greater dyspnea than those with oPIF as measured using the modified Medical Research Council scoring (mean (95% CI): 2.1 (2.0–2.2) vs 1.6 (1.4–1.7); *P*  < 0.001) and baseline dyspnea index (mean (95% CI): 5.1 (4.9–5.4) vs 6.1 (5.8–6.3); *P*  < 0.001). Based on COPD Assessment Test scores, participants with sPIF had a higher COPD symptom burden than those with oPIF (mean (95% CI): 21.5 (19.7–23.3) vs 19.5 (18.6–20.4); *P* = 0.05).

**Conclusion:**

In these trials, participants with COPD who had sPIF against the medium-low resistance DPIs had more dyspnea and worse health status than those with oPIF. These results demonstrate that sPIF is associated with a higher clinical burden as measured by patient-reported outcomes.

## 1. Introduction

Treatment with inhaled bronchodilators is the foundation of pharmacologic management of symptoms in patients with chronic obstructive pulmonary disease (COPD) [1]. Dry powder inhalers (DPIs), pressurized metered-dose inhalers (pMDIs), soft mist inhalers (SMIs), and nebulizers are the most commonly prescribed inhalation devices for the delivery of bronchodilators [2]. Each device requires a unique inhalation technique for optimal delivery of medication to the lower respiratory tract [3]. For optimal use of DPIs, patients must be able to generate sufficient peak inspiratory flow (PIF) against the internal resistance of the device to disaggregate powdered drugs into fine particles for lung deposition [4]. However, many patients with COPD have a suboptimal PIF (sPIF) and may not derive optimal benefit from DPIs. In observational studies, sPIF was observed in 19%–78% of stable outpatients with COPD and 32%–52% of inpatients before hospital discharge after treatment for COPD exacerbation [5–10].

sPIF in patients with COPD is associated with female sex, older age, shorter height, and lung function impairment [5–7, 9–11]. Low forced vital capacity (FVC) percent predicted and inspiratory capacity (IC) percent predicted are independent predictors of sPIF [9]. Despite several studies demonstrating the effects of age, sex, lung function parameters, and device resistance on PIF in patients with COPD, little is known about the association between inspiratory flow and severity of dyspnea and respiratory health status. In this analysis, we assessed the demographics and baseline clinical characteristics of participants with moderate-to-severe COPD from two randomized, controlled phase 3 trials of revefenacin [12–14] according to their PIF status (sPIF vs optimal PIF (oPIF)) to compare the population differences between the PIF subgroups. We used the optimal PIF of medium-low resistance DPIs such as Diskus®, Diskhaler®, and Ellipta® (60 L/min) [15, 16] as a cut-off value for these analyses because medium-low resistance has been used most frequently for reporting the prevalence of sPIF [7–10, 17, 18] and a PIF of ≥60 L/min is generally considered optimal for most DPI devices [7, 9, 17, 19].

## 2. Materials and Methods

### 2.1. Trial Design

Demographics and baseline clinical characteristics of participants with COPD were pooled from two randomized, controlled phase 3 trials, 0128 and 0149; both have been described previously [12–14]. In brief, trial 0128 (NCT02518139) was a 52-week, tiotropium-controlled, parallel-group phase 3 safety trial evaluating the safety and tolerability of revefenacin for nebulization in participants with moderate-to-very severe COPD [12, 13]. Trial 0149 (NCT03095456) was a 28-day, double-blind, double-dummy, parallel-group phase 3b trial comparing the effect of once-daily revefenacin for nebulization administered via the PARI LC® Sprint jet nebulizer with tiotropium administered via HandiHaler® on lung function in participants with moderate-to-very severe COPD and a PIF of <60 L/min against the medium-low resistance DPIs [14]. The trials were conducted in accordance with the principles of the International Council for Harmonisation of Technical Requirements for Pharmaceuticals for Human Use guidelines for good clinical practice and the code of ethics of the World Medical Association's Declaration of Helsinki, and all patients provided written informed consent.

### 2.2. Participants

Both trials enrolled participants diagnosed with moderate-to-severe COPD. Eligible participants had a smoking history of ≥10 packs per year, a postipratropium forced expiratory volume in 1 second (FEV_1_) to a FVC ratio of <0.7 at screening, and a postipratropium FEV_1_ of <80% of predicted normal and >700 mL at screening in trial 0128 and >400 mL in trial 0149. In addition, participants in trial 0149 had a PIF of <60 L/min.

### 2.3. PIF and Pulmonary Function Measurements

Baseline PIF was measured using the In-Check™ DIAL device (Alliance Tech Medical, Inc.) set to medium-low resistance DPI (R-2) and high resistance DPI (R-5) in trial 0149 and to R-5 alone in trial 0128. To measure PIF, participants were instructed to exhale completely, place the mouthpiece of the device into their mouths, and inhale as forcefully and deeply as possible. Participants repeated the PIF maneuver three times after adequate rest and recovery from each effort, with their PIF measurement reflecting the highest recorded value. Measurements were conducted at zero resistance followed by R-5 resistance in trial 0128 and at R-2 resistance followed by R-5 resistance in trial 0149. Data from trial 0149 were used to develop an algorithm to predictively correlate resistance in the R-2 device to resistance in the R-5 device. These values were then used to define PIF against the R-2 device in trial 0128. The methodology used to define the correlation between PIF against the R-2 device and PIF against the R-5 device has been described previously [20]. On the basis of this correlation analysis, a PIF value of 40 L/min against the R-5 device is approximately equivalent to a PIF of 60 L/min against the R-2 device [20]. oPIF against the resistance of the R-2 device was defined as >60 L/min, and sPIF was defined as ≤60 L/min.

Baseline lung function was evaluated by spirometric measurements of FEV_1_ and FVC. The distribution of the Global Initiative for Chronic Obstructive Lung Disease (GOLD) airflow limitation categories (GOLD 1, FEV_1_ ≥80% predicted (mild airflow obstruction); GOLD 2, FEV_1_ 50%–79% predicted (moderate airflow obstruction); GOLD 3, FEV_1_ 30%–49% predicted (severe airflow obstruction); and GOLD 4, FEV_1_ <30% predicted (very severe airflow obstruction)) between the PIF subgroups was also assessed.

### 2.4. Patient-Reported Outcomes

Dyspnea at baseline was assessed using the modified Medical Research Council (mMRC) dyspnea scale and the baseline dyspnea index (BDI) using standard methods [21–24]. A higher score on the mMRC scale and a lower score on the BDI represented greater dyspnea. An mMRC score of ≥2 was used as a threshold for distinguishing participants with more dyspnea from those with less dyspnea [1].

Participants' quality of life was assessed using the COPD Assessment Test (CAT) and St. George's Respiratory Questionnaire (SGRQ) [25, 26]. Participants with a CAT score of ≥10 were categorized as symptomatic [27] and those with a CAT score of ≥20 [1] as having more severe COPD symptoms. Participants with an SGRQ score of ≥40 were considered to have severe COPD symptoms [28]. CAT and SGRQ scores are reported only for trial 0128, as they were not assessed during trial 0149.

### 2.5. Statistical Analysis

Baseline characteristics, such as age, time since COPD diagnosis, smoking duration, height, weight, body mass index (BMI), PIF, percent predicted FEV_1_ and FVC, SGRQ score, and CAT score, are reported as mean values with 95% confidence intervals. Differences between the oPIF and sPIF subgroups were compared using a two-sample *t*-test.

## 3. Results

### 3.1. Baseline Demographics and Clinical Characteristics

Of the total number of participants enrolled, PIF data were available for 525 participants (actual measurements from 206 participants enrolled in trial 0149 and derived values for participants enrolled in trial 0128). Of these participants, 273 (52.0%) had sPIF (mean (95% CI): 44.6 L/min (43.4–45.8 L/min)), and 252 (48.0%) had oPIF (96.7 L/min (94.2–99.1 L/min)). Baseline characteristics of participants with optimal and suboptimal PIF against the medium-low resistance DPIs are presented in Table 1. Most participants were White and male; the mean age was 65 years. The number of participants who smoked, used concomitant inhaled corticosteroids/long-acting* β*-agonist combination treatment, and had at least one exacerbation in the year prior to trial initiation were consistent between the two subgroups. There were significant differences in height, weight, BMI, COPD duration, and smoking history between participants with oPIF and those with sPIF against medium-low resistance DPIs. Participants with sPIF were shorter and had a lower weight and BMI than participants with oPIF. They also had a longer COPD duration and smoking history than participants with oPIF.

### 3.2. Dyspnea Measures

**Figure 1:**
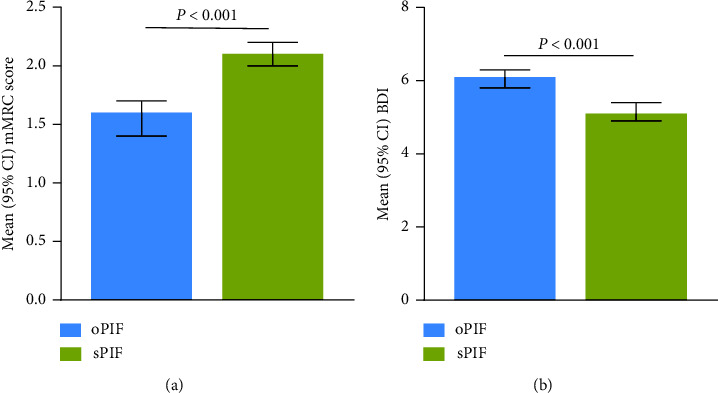
Mean (a) mMRC score and (b) BDI in participants with oPIF and sPIF against the medium-low resistance DPIs. BDI = baseline dyspnea index; CI = confidence interval; DPI = dry powder inhaler; mMRC = modified Medical Research Council; oPIF = optimal PIF; PIF = peak inspiratory flow; sPIF = suboptimal PIF.

Participants with sPIF had significantly greater dyspnea than participants with oPIF based on the mMRC score (*P* < 0.001; Figure 1(a); Table 2) and BDI (*P* < 0.001; Figure 1(b); Table 2). A significantly greater number of participants with sPIF than with oPIF had a mMRC of ≥2 (severe dyspnea; 70.0% vs 48.8%; *P*  < 0.001) and used supplemental oxygen (22.3% vs 9.5%; *P*  < 0.001).

### 3.3. Quality-of-Life Assessments

Of the 318 participants (250 participants with oPIF and 68 with sPIF) in trial 0128 with CAT and SGRQ scores, 64 participants (94.1%) with sPIF and 227 (90.8%) with oPIF had CAT scores of ≥10 (symptomatic). Forty-three participants (63.2%) with sPIF and 132 participants with oPIF (52.8%) had CAT scores of ≥20 (highly symptomatic). SGRQ scores of ≥40 were reported in 53 participants (77.9%) with sPIF and 180 participants (72.0%) with oPIF.

**Figure 2:**
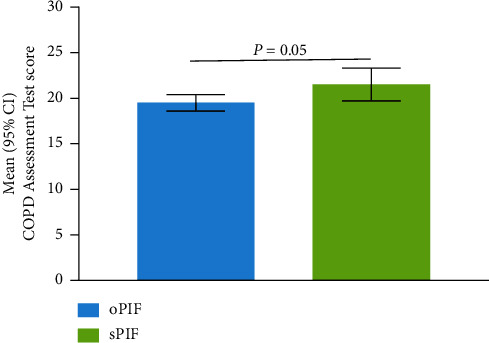
Mean CAT scores in participants with oPIF and sPIF against the medium-low resistance DPIs. CAT = COPD assessment test; CI = confidence interval; DPI = dry powder inhaler; oPIF = optimal PIF; PIF = peak inspiratory flow; sPIF = suboptimal PIF.

Participants with sPIF had a significantly higher COPD symptom burden than participants with oPIF on the basis of CAT's total score (*P*=0.05; Figure 2; Table 2). There was no significant difference in SGRQ scores between participants with sPIF and those with oPIF (*P*=0.22; Table 2).

### 3.4. Pulmonary Function Test

**Figure 3:**
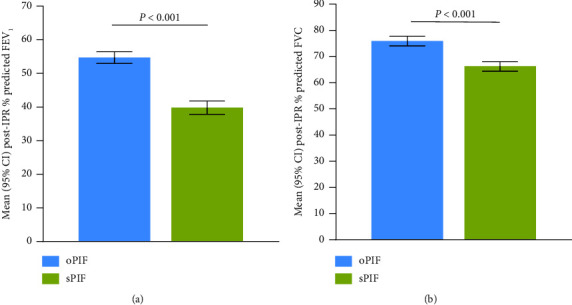
Mean percent predicted (a) FEV_1_ and (b) FVC in participants with oPIF and sPIF against the medium-low resistance DPIs. CI = confidence interval; FEV_1_ = forced expiratory volume in 1 second; FVC = forced vital capacity; IPR = ipratropium; oPIF = optimal PIF; PIF = peak inspiratory flow; sPIF = suboptimal PIF.

More participants with sPIF (41.4%) had very severe airflow obstruction (FEV_1_ <30% predicted) than participants with oPIF (6.0%; Figure S1). In comparison with oPIF, participants with sPIF had a significantly lower postipratropium percent predicted FEV_1_ (*P*  < 0.001; Figure 3(a); Table 2) and FVC (*P*  < 0.001; Figure 3(b); Table 2).

## 4. Discussion

In this analysis of data from two phase 3 trials of participants with moderate-to-very severe COPD, we have demonstrated that sPIF in patients with COPD is associated with high levels of dyspnea. Chronic dyspnea is one of the most common symptoms of COPD [1] and may be caused by a variety of mechanisms, including increased ventilatory demand, dynamic airway compression, lung hyperinflation, and respiratory muscle weakness [29]. PIF is determined by the patient's inspiratory effort and the strength of the inspiratory muscles [30]. Janssens and colleagues have demonstrated a significant correlation between PIF and both the inspiratory and expiratory mouth pressures, measures of respiratory muscle strength [7]. Respiratory muscle function is often compromised in COPD because of lung hyperinflation, hypoxemia, and muscle wasting [8]. Lung hyperinflation can affect PIF by shortening the vertical muscle fibers of the diaphragm, which in turn reduces the inspiratory muscle strength, and by adding an elastic load that must be overcome during inspiration [30, 31]. In addition, weight loss caused by poor nutrition and muscle wasting in patients with COPD can also lead to lower respiratory and peripheral muscle strength [32–35], resulting in dyspnea and sPIF. Thus, reduced inspiratory muscle strength in patients with COPD may be the common mechanism responsible for sPIF and dyspnea.

In addition to experiencing greater dyspnea, participants with sPIF against the medium-low resistance DPIs also had a higher perceived symptom burden than those with oPIF, on the basis of participants' CAT scores. More participants with sPIF than with oPIF reported SGRQ and CAT scores above the threshold for the disease with uncontrolled symptoms. SGRQ and CAT provide a comprehensive assessment of the COPD-specific health status of patients [1]; therefore, a significant difference between CAT scores in participants with sPIF and those with oPIF suggests that suboptimal PIF may be generally associated with poor health status. A significant association between sPIF and high levels of dyspnea and poor COPD-related health status was also recently demonstrated in outpatients with stable moderate-to-very severe COPD and sPIF [36].

In this analysis, participants with sPIF had a significantly lower FEV_1_ percent predicted and FVC percent predicted than did participants with oPIF. Other studies have not shown a consistent difference in spirometric measurements (FEV_1_ and FEV_1_ percent predicted) between participants with sPIF and oPIF [7–9, 11, 37], although Price and colleagues reported a weak correlation between FEV_1_ and PIF among participants who were discharged after hospitalization for a COPD exacerbation in a small retrospective observational study [38]. Results of another observational study that included 213 participants with advanced COPD also demonstrated significantly lower values for FVC percent predicted and IC percent predicted, but not for FEV_1_ percent predicted, in participants with sPIF (defined as <60 L/min against the simulated resistance of Diskus, a medium-low DPI) when compared with those with oPIF (≥60 L/min against the simulated resistance of Diskus) [9]. The differences in FVC percent predicted and IC percent predicted could be due to the greater air trapping and hyperinflation. Alternatively, lower lung volumes may be a result of lower inspiratory effort [9].

This analysis demonstrated that participants with sPIF had a significantly lower height, weight, and BMI, longer smoking history and COPD duration, and significantly more severe airflow obstruction than participants with oPIF. Previous studies have also shown that characteristics such as age, sex (female), and markers of hyperinflation are consistently associated with the presence of sPIF [5–7, 9, 11, 39]. Height and measures of lung function such as FVC percent predicted and IC percent predicted may also be associated with the presence of sPIF [5, 9]. Thus, our results generally support the published data.

This analysis has some limitations. In trial 0149, only participants with sPIF were enrolled; therefore, an estimate of prevalence cannot be provided from this analysis. Data for CAT and SGRQ tests were collected only in trial 0128, leading to considerably fewer participants in the sPIF subgroup than in the oPIF subgroup. Peak inspiratory flow against the resistance of medium-low resistance DPIs was measured only in trial 0149; in trial 0128, these values were estimated using the predictive model correlating PIF with medium-low resistance DPIs and high resistance DPIs from trial 0149 [20]. An additional limitation is that the trials on which this analysis was based had strict inclusion and exclusion criteria (e.g., exclusion of patients with significant comorbid pulmonary conditions or, for trial 0128, elevated cardiovascular risk) [12–14], and the trial population may therefore not be representative of the real-world population with COPD.

According to the 2024 GOLD strategy report, an individualized assessment of each patient's symptoms and future risk of exacerbations should be made before prescribing treatment for COPD [1]. Discovering measures and approaches that predict readmission following COPD exacerbation is needed to improve patient health. One study found that reduced PIF rate at discharge, a higher CAT score at discharge, frailty, and previous exacerbations were associated with hospital readmissions in patients with COPD [40]. Although some studies have found no relationship between the likelihood of readmission and the presence of sPIF among participants hospitalized for an acute COPD exacerbation [10, 41], others have shown that participants with sPIF are at increased risk of readmission [8]. Specifically, one retrospective analysis of patients who were hospitalized for acute exacerbation of COPD found that sPIF was common in these patients and sPIF predicted all-cause and COPD readmissions [8]. Participants with sPIF may not receive an adequate dose of bronchodilators through DPI devices to ameliorate their symptoms; therefore, in addition to measuring airflow limitation by spirometry, a healthcare provider should consider measuring PIF against the simulated resistance of the DPI before prescribing treatment. It has been suggested that PIF measured against the simulated resistance of a specific DPI may be used as a biomarker to identify patients who are likely or not likely to benefit from the DPI [42, 43]. In addition, characteristics such as sex, age, height, weight, BMI, and the markers of hyperinflation may also have utility in identifying participants at a higher risk for sPIF. Patients with sPIF are less likely to have a favorable response to DPIs and may be candidates for bronchodilator therapies administered via delivery systems that require low inspiratory efforts, such as pMDI, SMIs, and nebulizers.

In conclusion, in this analysis of pooled data from two phase 3 clinical trials, participants with COPD who had sPIF had significantly more dyspnea and worse health status than patients with oPIF, suggesting that sPIF is associated with a higher clinical burden than oPIF.

## Figures and Tables

**Table 1:** Demographics and baseline characteristics of participants according to PIF against the medium-low resistance DPIs.

Characteristics	oPIF^*∗*^ (*n* = 252)	sPIF^*∗*^ (*n* = 273)	*P* value
Age, mean (95% CI), y	64.6 (63.5–65.6)	65.4 (64.4–66.4)	0.25
Sex, male, *n* (%)	167 (66.3)	162 (59.3)	0.10
Race, White, *n* (%)	235 (93.3)	248 (90.8)	—
Weight, mean (95% CI), kg	86.7 (84.1–89.3)	80.5 (78.0–83.0)	<0.001
Height, mean (95% CI), cm	172.7 (171.6–173.8)	169.9 (168.8–171.1)	<0.001
BMI, mean (95% CI), kg^2^/cm	29.0 (28.2–29.7)	27.8 (27.0–28.6)	0.04
Current smoker, *n* (%)	114 (45.2)	127 (46.5)	0.77
Smoking duration, mean (95% CI), y	39.2 (37.8–40.5)	41.3 (40.0–42.5)	0.02
Duration of COPD diagnosis, mean (95% CI), y	9.0 (8.3–9.8)	10.8 (10.0–11.5)	0.002
Concurrent LABA or ICS/LABA use, *n* (%)	139 (55.2)	149 (54.6)	0.89
PIF, mean (95% CI), L/min	96.7 (94.2–99.1)	44.6 (43.4–45.8)	<0.001^†^
Participants with ≥1 exacerbation in the prior year, *n* (%)	61 (24.2)	81 (29.7)	0.16

^*∗*^oPIF was defined as PIF >60 L/min and sPIF as PIF ≤60 L/min. ^†^The difference in baseline PIF between the subgroups was significant because all participants from trial 0149 had sPIF (<60 L/min). BMI = body mass index; CI = confidence interval; COPD = chronic obstructive pulmonary disease; DPI = dry powder inhaler; ICS = inhaled corticosteroid; LABA = long-acting *β*-agonist; oPIF = optimal PIF; PIF = peak inspiratory flow; sPIF = suboptimal PIF.

**Table 2:** Summary of dyspnea measures, health status, and pulmonary function test in participants with oPIF and sPIF against the medium-low resistance DPIs.

	oPIF^*∗*^	sPIF^*∗*^	*P* value
mMRC score, mean (95% CI [*n*])	1.6 (1.4–1.7 [250])	2.1 (2.0–2.2 [273])	<0.001
BDI, mean (95% CI [*n*])	6.1 (5.8–6.3 [234])	5.1 (4.9–5.4 [272])	<0.001
Total CAT score, mean (95% CI [*n*])	19.5 (18.6–20.4 [250])	21.5 (19.7–23.3 [68])	0.05
SGRQ score, mean (95% CI [*n*])	50.0 (47.9–52.1 [250])	52.8 (48.8–56.8 [68])	0.22
Post-IPR percent predicted FEV_1_, mean (95% CI [*n*])	54.7 (52.9–56.5 [252])	39.8 (37.9–41.8 [273])	<0.001
Post-IPR percent predicted FVC, mean (95% CI [*n*])	75.9 (74.1–77.7 [252])	66.2 (64.4–68.0 [273])	<0.001

^*∗*^oPIF was defined as PIF >60 L/min and sPIF as PIF ≤60 L/min. BDI = baseline dyspnea index; CAT = COPD Assessment Test; CI = confidence interval; DPI = dry powder inhaler; FEV_1_ = forced expiratory volume in 1 second; FVC = forced vital capacity; IPR = ipratropium; mMRC = modified Medical Research Council; oPIF = optimal PIF; PIF = peak inspiratory flow; SGRQ = St. George's Respiratory Questionnaire; sPIF = suboptimal PIF.

## Data Availability

Summary data for all analyses described in this paper are presented within the results of the submitted manuscript, including in tables/graphs. To protect the privacy of trial participants and investigators, Theravance Biopharma (and its affiliates) does not share individual deidentified participant data or other relevant trial documents.

## Supplementary Materials

Supplementary Materials Figure S1: distribution of airflow limitation categories in patients with oPIF and sPIF against the simulated medium-low resistance in trials 0128 and 0149. *Graphical Abstract*: In this analysis of data from two phase 3 clinical trials of participants with moderate-to-very severe chronic obstructive pulmonary disease (COPD), the authors demonstrated that suboptimal peak inspiratory flow (sPIF) is associated with significantly more dyspnea as measured by the (A) modified Medical Research Council (mMRC) scoring and (B) the baseline dyspnea index (BDI). Compared with optimal PIF (oPIF), sPIF was associated with a higher clinical burden of COPD.
