# Supplementary material for: Clinical Burden of Chronic Obstructive Pulmonary Disease in Patients with Suboptimal Peak Inspiratory Flow
Source: Can Respir J. 2024 Mar 22;2024:8034923. doi: 10.1155/2024/8034923 (PMC10980549; doi:10.1155/2024/8034923)
Supplement: Supplementary Materials — Figure S1: distribution of airflow limitation categories in patients with oPIF and sPIF against the simulated medium-low resistance in trials 0128 and 0149. Graphical Abstract: In this analysis of data from two phase 3 clinical trials of participants with moderate-to-very severe chronic obstructive pulmonary disease (COPD), the authors demonstrated that suboptimal peak inspiratory flow (sPIF) is associated with significantly more dyspnea as measured by the (A) modified Medical Research Council (mMRC) scoring and (B) the baseline dyspnea index (BDI). Compared with optimal PIF (oPIF), sPIF was associated with a higher clinical burden of COPD. [file 8034923.f1.zip › Ohar_COPD Burden Manuscript_graphical abstract.pdf]

**Graphical Abstract**

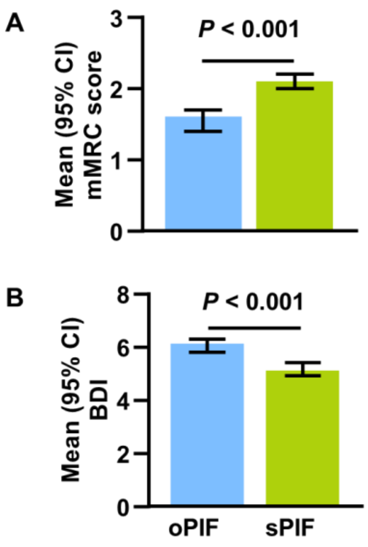


In this analysis of data from two phase 3 clinical trials of participants with moderate to very severe chronic obstructive pulmonary disease (COPD), we demonstrated that suboptimal peak inspiratory flow (sPIF) is associated with significantly more dyspnea as measured by (A) modified Medical Research Council (mMRC) scoring and (B) the baseline dyspnea index (BDI). Compared with optimal PIF (oPIF), sPIF was associated with a higher clinical burden of COPD.
